# Improving Collaboration Between Primary and Secondary Mental Healthcare via Boundary Spanning: Evaluation of a New Joined‐Up Community Mental Healthcare Model in England

**DOI:** 10.1002/hpm.3949

**Published:** 2025-05-22

**Authors:** Lida Efstathopoulou, Jules Mackenzie, Rory Cameron, Adam P. Wagner, Julia Jones, Jesus Perez

**Affiliations:** ^1^ Cambridgeshire and Peterborough NHS Foundation Trust Fulbourn UK; ^2^ Centre for Research in Public Health and Community Care University of Hertfordshire Hatfield UK; ^3^ NIHR Applied Research Collaboration East of England Cambridge UK; ^4^ Norwich Medical School University of East Anglia Norwich UK; ^5^ Department of Psychiatry University of Cambridge Cambridge UK; ^6^ Institute of Biomedical Research of Salamanca University of Salamanca Salamanca Spain

**Keywords:** care integration, community mental healthcare, primary care

## Abstract

**Objectives:**

Community mental healthcare requires the collaboration of multiple services to meet the needs of local populations. Accessing mental health care in England often involves the collaboration of primary and secondary healthcare services. This paper presents the findings from an evaluation of ‘boundary spanning’ processes and practitioner roles aiming to reduce service fragmentation and improve access to mental healthcare.

**Methods:**

Forty‐one qualitative interviews with professionals across local healthcare providers were conducted in Peterborough (East England) to assess the impact of boundary spanning processes and practitioner roles and were analysed thematically.

**Results:**

Structured boundary spanning processes and professional roles were found to facilitate communication and knowledge exchange between primary and secondary mental healthcare services, leading to optimisation of GPs' decisions about individuals' treatment pathways, and to improvements in service accessibility. Yet, effectiveness was reported as conditional on GPs' engagement, as well as the decentralised structure of primary care settings.

**Conclusion:**

Community mental healthcare organisations could utilise boundary spanning interventions to flex organisational barriers between primary and mental healthcare and optimise accessibility of service users to mental health services. Boundary spanning processes and professional roles can be used to inform national and local care integration strategies.

**Abbreviations**
CPFT: Cambridgeshire and Peterborough NHS Foundation Trust
CPSL MIND: Cambridgeshire, Peterborough, and South Lincolnshire (CPSL) MIND
DNA: Did Not Attend
EUPD: Emotionally Unstable Personality Disorders
GP: General Practitioner
GPN: Greater Peterborough Network
HRA: Health Research Authority
MDT: Multidisciplinary Team
MHLPs: Mental Health Lead Practitioners
NHS: National Health Service
NIHR: National Institute for Health Research
PCMHS: Primary care mental health service
PCNs: Primary Care Network
PWS: Psychological Wellbeing Service
VC: Virtual Clinic

**Summary**

- ‘Boundary spanning’ practices enable knowledge exchange between primary care and secondary mental healthcare
- Improving knowledge exchange among professionals can improve access of patients to mental health services
- ‘Boundary spanning’ can be a useful approach for care integration
- While professional diversity enables to holistically support patients' needs, it may become a restraining factor to the success of knowledge exchange interventions

## 1 Introduction

Community mental healthcare is defined as a range of generic and specialist services providing mental health treatment or support to individuals within their community, in combination with hospital care [1, 2]. Such mental healthcare models emphasise the need for service provision in communities where people live, whereas historically most mental health treatment has been provided in hospital settings [2]. This healthcare approach is found in high income countries, where resources allow for the development of services targeting different mental health conditions, comorbidity needs, and short‐ and long‐term treatment options [2]. Community mental healthcare models are based on a patient‐centred approach, where services offered are formed around individuals' needs and provided collaboratively by health, social care, third sector and other service providers [1, 3].

Principles of system collaboration governing community mental health care stem from care integration approaches. Existing studies have assessed the impact of integration approaches on care provision, including its impact on service accessibility, patient outcomes, as well as its economic impact [4, 5, 6]. Further research is still needed to identify processes that enable care integration, considering the role of the inter‐organisational context and principles of communication and collaboration among service providers when developing inter‐organisational relationships [4, 5, 6]. This paper contributes to exploring the impact of ‘boundary spanning’ roles on inter‐organisational relationships in healthcare, an approach focusing on reducing organisational barriers by adopting knowledge exchange and collaboration practices [7]. Findings presented in this paper are from the evaluation of a community mental healthcare model implemented in one county in England and add to existing literature of the role of boundary spanning in enabling healthcare delivery, with a focus on the interface between primary and mental healthcare.

In England, principles of care integration are acknowledged as having a key role on the delivery of community mental healthcare [8]. Collaboration among service providers, however, is often disconnected, particularly when it comes to patients' transitioning from primary healthcare to secondary mental healthcare [9]. Primary care is organised in General Practices (GPs) that operate within primary care collaborations, called Primary Care Networks (PCNs), responsible for meeting the primary healthcare needs of local populations [10]. Access from primary care to specialist mental health services is managed by referring primary care patients to secondary mental healthcare services, provided by mental health organisations (also called ‘Trusts’). Patients with complex mental health needs, however, frequently face long waiting times to access or are not accepted to specialist mental healthcare services due high access thresholds [11]. At the same time, they may also not be accepted into psychological therapy services (called ‘Improving Access to Psychological Therapies’ (IAPT)), if their needs are considered too complex, falling into a gap existing between primary and secondary care [11]. As a result, a lack of clarity and capacity in care pathways between primary and mental healthcare services become barriers to meeting the range of mental health needs presented in primary care.

Barriers to accessing specialist mental health services combined with rising demand for mental health support in primary care have increased the pressure on GPs [12]. In England, GP referrals to secondary mental healthcare have increased by approximately 20% within a 5‐year period prior to the COVID‐10 pandemic [11]. The COVID‐19 pandemic has significantly added to the amount and complexity of mental health needs presenting in primary care [13]. Overall contacts with secondary mental healthcare services have increased by 24% following the pandemic, augmenting the demand pressure as a secondary care level [14] and likely leading to higher thresholds for primary care referrals. To address service disparities and increasing pressure for accessing mental health services, national policies highlight the need for new community mental healthcare models that improve the collaboration between primary and mental healthcare and facilitate access to mental health support and treatment [8, 15].

Effective collaboration between health and care providers requires both the establishment of inter‐organisational processes and investment in knowledge sharing among diverse professional groups [16]. A useful way to improve collaboration is by investing in ‘boundary spanning’. Boundary‐spanning refers to sets of activities delivered by individuals with the aim to facilitate knowledge exchange by developing intra‐ or inter‐organisational relationships [17]. Boundary spanners are professionals working on the interface of services or organisations to enable communication and address barriers that restrict the mobilisation of knowledge [7]. They can also be researchers transferring research knowledge to policy or practice, while enable learning from organisational settings or policy makers to researchers [18, 19, 20].

Effective boundary spanners have a versatile skillset, including networking skills, ability to develop trust relationships with partners, and understanding new organisational and professional cultures [7]. Their ability to synthesise new knowledge and use it for decision‐making purposes are key attributes to this role [7]. Emphasis is also given to their ability to understand diverse audiences and cultural differences among organisations [21]. Boundary spanners are also called to deal with and manage complexity around inter‐organisational relationships and contradicting interests that may exist between organisations [22]^.^

Initially developed within organisation and management literature, boundary spanning has been studied within private and public organisations with a local, national or international remit to understand its contribution in establishing knowledge exchange pathways between teams, departments or organisations [21, 23]. For example, in international private corporations, boundary spanners play a crucial role in coordinating knowledge exchange between global region departments and partners [24]. At a local level in the public sphere, boundary spanners can be acts as key players in knowledge exchange between communities and organisations and influence the types of interventions implemented [21]. To address knowledge exchange needs at different organisational levels, boundary spanners can hold roles from senior management to practitioners [22].

Interest about boundary spanning has increased within the public sector, particularly in healthcare, where such concepts are seen as facilitators to the interface between health and care providers and the delivery of joined‐up care pathways [22, 25]. Boundary spanning in healthcare focuses on knowledge exchange among organisations or professional groups, with the purpose of enabling collaboration among those working in silos [26]. Here, the purpose of boundary spanning is to flex organisational barriers that have been found to inhibit timely access to care [26].

Boundary spanners work close to their organisation's boundaries and where there are connections with other organisations, and use their knowledge and expertise, as well as communication and networking skills, to facilitate joined working [16]. They usually hold expertise on a specific health or care subject, for example, specialist nursing, knowledge which they also aim to share across their organisation's boundaries as part of their role [26]. Boundary spanners are also equipped with knowledge of the wider health and care system within which they operate, allowing them to operate as knowledge brokers and relationships builders [17]. [7]Boundary spanning roles have already been implemented within the UK's healthcare system, including advisory rehabilitation roles, human immunodeficiency virus (HIV) nurse coordinators, domestic violence and abuse workers and care navigators [16, 26, 27].

Evaluations of boundary‐spanning roles suggest they contribute to improved relationships within primary care, between the public healthcare the private sector, better care coordination among healthcare providers, and decreased fragmentation among services [26, 27, 28]. Less is known, however, about the potential contribution of boundary spanning to improving care pathways between primary and mental healthcare services. While several studies have assessed the role of individual boundary spanners, inter‐organisational processes of boundary spanning have received less attention [25].

This paper presents findings from the service evaluation of the ‘Peterborough Exemplar’, an NHS community mental healthcare model implemented in Peterborough (East England) about the contribution of (a) boundary spanning professional roles to addressing service fragmentation and barriers to accessing mental healthcare, and (b) boundary spanning structure processes, as a shared space bringing together professionals from primary and secondary mental healthcare.

### 1.1 The Peterborough Exemplar

The Peterborough Exemplar is a community mental healthcare model, and one of the 12 early implementer models funded by NHS England aiming to enhance mental health service provision within communities and strengthen care integration [8]. The intervention was implemented in Peterborough (2020–2021) at the north part of the Peterborough and Cambridgeshire Country (England). Peterborough was identified as an area in need for improved service provision by the Exemplar Partners due to deprivation [29]. The structure of the Exemplar model was informed by local population and service provision data, as well as Exemplar stakeholders' expertise [29].

The Exemplar partners are mental health, social care and third sector local service providers supporting the mental health needs of the population in the Peterborough and Cambridgeshire Country and they were commissioned to design and co‐deliver the Peterborough Exemplar (Cambridgeshire and Peterborough NHS Foundation Trust (CPFT), Cambridgeshire and Peterborough Clinical Commissioning Group (CCG), Cambridgeshire, Peterborough, and South Lincolnshire (CPSL) MIND charity, Peterborough City Council, SUN Network, and the Greater Peterborough Network (GPN), a coalition of General Practices) [29].

The full structure of the intervention is described in detail in the evaluation's protocol [29]. In summary, the Exemplar is a complex intervention, entailing different organisational and inter‐organisational components related to each other [29, 30]. The intervention is based on two main pillars:

- *Improving relationships between primary care and secondary mental healthcare, as well as the local community.* This component focused on:
  - Improving the collaboration between GPs and mental health professionals, by introducing:
    1. *Structured inter‐organisational processes*: two types of routine meetings between mental health professionals (e.g., nurses, psychiatrists) and GPs, aiming to address (1) individual service user needs and (2) potential improvements in care pathways. The meetings are part of the Primary Care Mental Health Service (PCMHS), a gatekeeping service for service users referred to secondary mental healthcare.
    2. *Liaison practitioners*: Mental health professionals offering advice to GPs and other health professionals about mental health needs and service provision. A key characteristic of liaison practitioners is that they have expertise in a specific mental health domain, for example, personality disorders or pharmacological management.
  - *Community engagement*: A team of professionals hosted by the mental health organisation, focusing on enhancing communication and information dissemination about NHS and non‐NHS interventions, and other available resources that can support service users' mental health and wellbeing. The team members engaged with organisations across the local community, including mental health, social care, third sector and other organisations, such as community groups or physical activity groups.
- *Introducing new mental health services*: Addressing the service gap between primary care and secondary mental healthcare by enhancing the Personality Disorders Community Mental Health Service offering treatment to people with moderate personality disorders. A number of new services were also created: the Psychological Skills Service offering brief psychological therapies, group therapies and recovery therapies; the Dual Diagnosis and Outreach Team, supporting people with dual diagnosis often being homeless, and the Social Care Team offering early interventions to individuals who need social care support (this service was offered in collaboration with local Adult Care Services having a county‐wide remit). The said services aim to support service users with complex mental health needs, who do not reach the threshold of secondary specialist mental healthcare teams.

**FIGURE 1:**
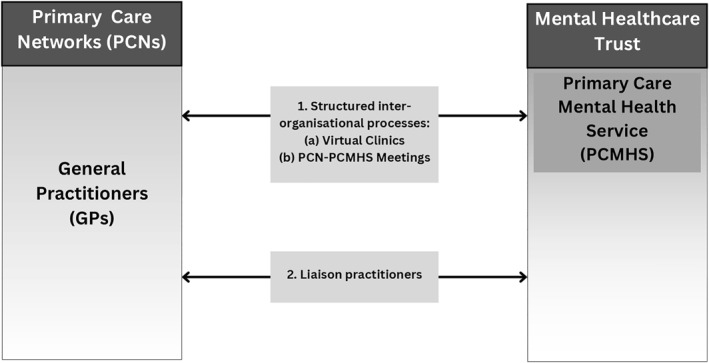
Components of the Peterborough Exemplar focusing on the interface between primary and secondary mental healthcare, that is, 1. Structured inter‐organisational processes, and 2. Liaison practitioners.

Findings presented in this paper focus on the first pillar of the Peterborough Exemplar, and specifically on the implications of the intervention on the collaboration between primary care and secondary mental healthcare. Primary care in the area of Peterborough in organised in Primary Care Networks, that is, collaborations of GPs covering the primary care needs of predefined geographic areas [10]. Secondary mental healthcare is provided by an NHS mental health organisation (called ‘Trust’) supporting the mental health needs of the local population by offering community and impatient mental health services [8]. The Figure 1 below presents the components of the Exemplar intervention specifically focusing on facilitative inter‐organisational relationships between primary and secondary mental healthcare presented in this paper, that is, structured inter‐organisational processes, and liaison practitioners (see Figure 1).

## 2 Methods

Qualitative findings presented in this paper are part of a larger service evaluation adopting a multi‐methods approach and employing quantitative methods and qualitative interviewing. The evaluation design had two main objectives: (a) to assess the intervention's impact on service provision and patient outcomes comparing the intervention group (Peterborough area) with a non‐equivalent comparator control group (Fenland area) selected based on population and deprivation indicators, and (b) the intervention's implementation, see protocol here [29]. Quantitative analysis will be reported separately in the future presenting the intervention's contribution on addressing service demand. Qualitative interviews focused on the evaluated implementation, including the impact of the intervention on the relationships among local service providers [29].

### 2.1 Qualitative Interviews

The Consolidated criteria for reporting qualitative research (COREQ) are used to report the data collection process, analysis and findings [31]. Qualitative semi‐structured interviews were conducted with health and care professionals employed by Exemplar partners. Qualitative interviewing allows a rich exploration of individual perspectives about a particular phenomenon. Here, we utilised a semi‐structured dialogue with a researcher, where participants offered insight into the discussed topic [32]. An interview guide was developed to lead the interviews [29, 33]. Key topics focused on (i) the implementation of the intervention, (ii) the role of inter‐organisational relationships and (iii) the role of patient‐centred approach. Two workshops with service users were held to receive recommendations about the evaluation design, including their experience with transitioning from primary to secondary mental healthcare. Outcomes of the workshops informed the interview guide of qualitative interviews. The guide can be found at the evaluation protocol [29].

### 2.2 Sampling and Recruitment

We employed purposeful sampling to identify potential participants. Purposeful sampling focuses on selecting individuals most relevant to the studied topic, aiming to provide valuable and rich insight into the questions discussed [34]. Representatives from different levels of care, services and Exemplar Partners were invited to participate in the interviews, including senior managers, managers, and frontline workers, to ensure representation. Most of the Exemplar services were deliver by the participating Trust, leading to higher number of interview participants. We also adopted a snowballing approach, where we asked interview participants to recommend colleagues that would be potentially interested in being interviewed [34]. Identified individuals received an invitation email, including a participant information sheet and a consent form. The participant information sheet included details about the study, the purpose of the study, and the process of delivering interviews by one individual researcher, data recording and the use of anonymised quotes. The table below (Table 1) shows the number of professionals planned to be included in the interview process and those who agreed to participate.

**TABLE 1:** Summary of professionals planned and accepted participating in qualitative interviewing.

Group	Professional group	Planned	Accepted	Organisation
Frontline workers—Primary care	Total	28	25	
GPs	GPs	8	4	Primary care
Exemplar services	CPFT staff	18	19	CPFT
	Third sector staff	2	2	Third sector
Frontline workers—CPFT secondary care	Total	6	5	
	CPFT clinicians/liaison roles	6	5	CPFT
Management	Total	7	6	
	Social care managers	1	1	Social care
	PCN^a^ mental health leads	2	1	Primary care
	Project managers	3	3	CPFT, CCG
	Managers (third sector)	1	1	MIND
Senior management	Total	6	5	
	Commissioners	2	2	CCG
	Senior management	2	2	Third sector
		2	1	CPFT
	Total of participants	47	41	

Abbreviation: PCN: primary care network.

^a^ Group of GP practices collaboratively covering primary care needs of a geographic area in England.

A total of 41 individual interviews were conducted via Microsoft (MS) Teams, generating 2071 recorded minutes (mean duration: 48 min). Interviews were conducted by the lead researcher (first author), who has 6 years of experience in delivering qualitative interviews and analysing qualitative data. Qualitative interviews were conducted from June 2021 to February 2022. No interviews were repeated. Interviews were recorded with an audio recorder or using Microsoft (MS) Teams, and transcribed verbatim. Transcript were returned to participants when requested; a tick‐box option for returning transcripts was included in the consent form. This option was available for participants who wish to hold a record of the transcript or make any changes; however, we did not receive any transcript corrections from interview participants.

### 2.3 Qualitative Analysis

Thematic analysis was used to identify key themes generated by summarising qualitative data under meaningful groups, that is, codes [35]. Analysis followed an inductive approach, where codes were generated by grouping textual data with similar meanings and then codes were grouped under themes. Following initial coding, codes identified were refined to improve the quality of coding, merged, or split where necessary [35]. This was an iterative process, where the lead researcher (first author) would refine codes throughout the coding process. Interview transcripts were coded by using the NVIVO data management programme [36]. After completing the coding process, another researcher independently coded a sample of textual data. Sample coding was then reviewed and compared with the initially coding framework by the lead researcher.

Ahead of data collection, we used the Health Research Authority (HRA) tool developed to verify if a study is research or evaluation; the study was confirmed as a service evaluation [37]. The service evaluation was approved by the Quality Improvement department of the Cambridgeshire and Peterborough NHS Foundation Trust (CPFT) and registered in the Trust's database (date: 06/05/21). Several steps were followed to ensure data collected were managed appropriately and in line with GDPR requirements. Qualitative interviews were stored in a password protected laptop, accessible only to the lead researcher. Recordings were deleted following transcription, and each transcript was attributed a code. Interview participants were informed that anonymised quotes will used in publications in the participant information sheet and consent form.

## 3 Findings

### 3.1 Boundary Spanning Processes Enable Knowledge Exchange

The interface between primary and mental health professionals is supported by two structured inter‐organisational processes. The ‘Virtual Clinics’ and ‘PCN Lead—PCMHS Lead meetings’ are held regularly (monthly unless decided otherwise) and focus on identifying solutions about (a) treatment of individual service users, and (b) clinical pathways. The impact of these processes was highlighted in the interviews:

- Virtual Clinics (VCs)

Evidence shows that VCs facilitate discussions about service users' mental health conditions and available services to which patients can be referred by the GPs. Specifically, outcomes of discussions during VCs are used to inform decision making about appropriate referrals of service users to mental health services or signposting to other community services. VCs are also used to support GPs with managing the care pathways of service users with complex needs who require more frequent support.

You can have that opportunity to discuss patients that, you know, might have come through as a referral. So actually, being able to discuss that, either before it comes through or actually encourage the referral to come through, if that makes sense. But also discuss a lot of patients that are quite complex. GPs might be spending a lot of time with these patients, within their clinics, and actually being able to offer advice and guidance regarding that, I think, has been really helpful.

CPFT frontline staff member (10)

Virtual Clinics were also identified as a valuable platform for mental health professionals to disseminate knowledge to GPs about the specific purpose of mental health services (e.g., what treatments are offered) and the referral criteria of each service.

it’s about services that are available that the person might benefit from that they probably haven’t heard of or had any contact with. […] GPs will refer a person that has EUPD [Emotionally Unstable Personality Disorders] to Psychological Wellbeing Service. So, that’s, sort of, delaying the patient as well because they are not going to be usually accepted, unless they’re very stable. So, it is, sort of, education around which service will accept symptoms or diagnosis.

CPFT frontline staff member (31)

b. PCN‐PCMHS Meetings

A PCN GP with leading responsibilities and a PCMHS senior mental health professional meet monthly to oversee the collaboration between PCMHS and GPs. Interviewees explained that meetings focused on the interface between primary and mental healthcare and on monitoring key service activity indicators. This space provides opportunities to discuss ‘issues and concerns’ from GPs [*General Practitioner (11)*], attendance at VCs, referral rates, or similar indicators.

I meet monthly with the GP PCN leads for [PCN 1] and [PCN 2]. We are thinking about improvements that will be within Exemplar, and how we can improve the engagement with the surgeries. That is around thinking about virtual clinics, thinking about, DNA [did not attend] rates within PCMHS, reviewing suicides, thinking about compliments and complaints, in terms of both services really, so that we are open in terms of how we think and how we can really work better together.

CPFT frontline staff member (10)

We have got someone to talk to about DNAs *[do not attend]* at the CPFT and how we can then manage that.

General Practitioner (11)

c. Informal communication between GPs and PCMHS mental health professionals

Mental health professionals explained that GPs also reach out in an informal way (e.g., email or phone call) and as needed to discuss patient cases, in addition to engaging with structured processes. Informal inter‐organisational communication was not part of the initial implemented inversion, yet findings suggest it was developed as an outcome of it. Such findings indicate an organic engagement of GPs to the PCMHS offer, where informal communication routes are utilised to advise decisions about service users' care pathways. Informal communication pathways are likely enhanced by professionals' interface via the structured meetings and mental health professionals' availability to offer support to GPs.

So, the GPs do tend to just email me. […] As soon as you get the first [GP practice], they phone, they ring, the consultant is in the virtual clinic, so that gives them… they can have medical reviews there and then, rather than just sending a patient through assessments, they can just get advice. So, it is really positive. […] So, just having that comfort, I think.

CPFT frontline staff member (20)

### 3.2 Outcomes of Boundary Spanning Processes

- Optimisation of decision‐making about individual service users

Analysis indicates that a key outcome of structured processes and informal communication pathways is the identification of appropriate care pathways for individual service users. Patient cases are discussed in informal or structured settings, such as VCs, with PCMHS professionals to identify appropriate treatment options, medication solutions or signposting to community support.

We get some advice on medication changes, which we can do. Either [PCMHS] say, ‘Actually this patient would be suitable to be referred in.’ I will type a quick referral saying, ‘Dear colleague, as discussed in clinic, please accept this referral.’ Just a very quick thing so that they can see the patient. Sometimes we might decide that, actually, mental health support is not exactly what these patients require. We might signpost them to other services.

General Practitioner (41)

These communication pathways also appear to allow for the discussion and resolution of accessibility barriers. Opportunities to discuss individuals' cases increase GPs' clarity about service user care status and resolve accessibility barriers for individual service users.

Other [GPs] will send me a task about somebody that they feel hasn't been sorted out well enough. They think they [i.e., patients] have just been discharged, but it has been trying to point out to them they have not been discharged. They have been put into a group or into a pathway that is there. Yes, so I think, again, it is just having that confidence to say, ‘No, it has been done really well. Look, this is what has happened.’

General Practitioner (43)

Furthermore, findings demonstrate that the involvement of mental health professionals in managing service users and supporting decisions around their care, particularly of service users with complex needs, can reduce GP concerns.

So, putting something in that provides that level of confidence about how best to manage those patients to prevent escalating risky behaviours. […] Putting some expertise in that space is probably very, very helpful for the system, for professionals as well as for that cohort of patients. Who are hopefully going to get support sooner rather than having to raise their risk in order to get through the next hoop of accessing support.

CPFT senior management staff member (28)

b. Optimisation of referral and clinical pathways

The findings suggest that structured and informal pathways between GPs and the PCMHS creates opportunities for the two parties to jointly discuss and resolve, where possible, repeating accessibility problems, for instance, the adoption a new practice (e.g., review the structure of referral forms). A General Practitioner explained that:

One of the issues that we brought up to [PCMHS Staff Member] was that our people who were referred, by the PCMHS, to the psychological wellbeing service (PWS), they were deemed too complicated for the psychological wellbeing service to deal with. […] We fed this back, and we came up with a plan that the PWS and the PCMHS have a monthly meeting where they can feed back any patients, they deem to be too complicated directly back to the PCMHS rather than involving us when we were not involved in the first place. That is one thing that has helped a lot.

General Practitioner (41)

### 3.3 Liaison Practitioners as Boundary Spanners

Participants argued that liaison practitioners contribute to managing service users with mental health needs by offering mental health expertise to GPs who use such knowledge to inform decisions about treatment. As with PCMHS mental health professionals, sharing of mental health expertise is also valuable for managing service users with complex needs who may reappear to GP practices due to difficulties engaging with services. As a result, the roles appear to strengthen interaction between the two parties and enable targeted referrals. Liaison practitioners engage with GPs either by participating in Virtual Clinics or via informal communication.

For me to feel that there is [Liaison Practitioner] that is really worth referring to, because I have engaged with them, I have spoken to them, I have seen what they say and how passionate they are about it, I can then pass that on to my colleagues. Then that is a really great way of getting the link between primary and secondary care.

General Practitioner (42)

Some liaison practitioners explained that they perceive active engagement with GPs as a key part of their job role and that it is an essential element for developing sustainable relationships with primary care. Practitioners appear to actively attempt to engage with GPs, offer support and resolve operational issues in care delivery.

Well, in order to do my job, I need to build up relationships with GPs and I need them to know who I am, I need them to feel that I’ve got something useful to say and that they can ask me for help. So, this is where the Primary Care Mental Health Service (PCMHS) are a great link because they obviously have set up the virtual clinics.

CPFT liaison practitioner (03)

### 3.4 Factors Inhibiting the Impact of Boundary Spanning

- Lack of clarity about mental health services and community support

The interview findings suggest factors that can inhibit the impact of boundary spanning practices. Data indicate that some GPs still have limited understanding about the core function of the PCMHS, the offer of specialist mental health services and support within the community. For instance, CPFT staff members and GPs explained there is not always good understanding of the referral process to the PCMHS or the purpose of Virtual Clinics.

But I appreciate there might be other pockets of areas where there may be still some work to be done. You can see sometimes some GPs are quite frustrated about what we can offer and misunderstanding, maybe, the role of the Mental Health Lead Practitioners (MHLPs).

CPFT senior management staff member (28)

b. Diverse professional views about treating patients with mental health challenges

Findings suggest that there is diversity in professional views among GPs about the value of available care options within the community and the contribution they can have on people's health. As a result, service users may miss opportunities to benefit from community mental health services and resources. Participants suggested that there is still limited understanding about the potential of non‐NHS mental health services and community resources in supporting individuals with mental health needs, leading to de‐prioritisation of non‐medical solutions for mental health. Some GPs' professional views are that optimal mental health treatment coming primarily from NHS mental health services, excluding other offers for mental health, for example, a recovery support worker in the third sector.

And I think that is where some of the frustration lies that GPs find with PCMHS, is that actually PCMHS might do the assessment and say, Actually, Mind [Mental health charity] will be really helpful in this. But actually, that does not necessarily validate the struggle that they might have had with that patient. So that medicalised view of, ‘If me as a GP can’t address it, a specialist medic could pick this up,’ as opposed to a support worker.

CPFT senior management staff member (28)

c. Discrepancy between new PCMHS investment and GPs' priorities

Interviews with GPs highlighted that the gatekeeping role of the PCMHS can be interpreted as additional workload, as it requires time from the GP to identify, discuss in VCs and refer patients to PCMHS or other mental health support within the community. This is in comparison to previous processes where GPs would refer directly to a mental health service, as described by one GP.

There is a potential for feeling that it is increasing, it is adding another layer rather than reducing workload for us. […] You are processing the information for someone else initially and then with someone else afterwards during the meeting. Then often the actions to do come back to us again, so you could argue it is tripling our work. […] I do not think it has saved us time. Not so far.

General Practitioner (11)

A GP highlighted the need to enhance support for service users in mental health crisis. While PCMHS professionals offer expertise for routine referrals, support is not equally efficient for service users in a crisis state. The NHS emergency phone line (called ‘First Response Service’) is used instead, which appears to be disconnected from mental health services, maintaining service fragmentation.

One of the issues with dealing with people with mental health problems is that we often must deal with crises. It would almost be nice to have something which I understand would be virtual, but almost like a virtual multidisciplinary team (MDT). I could refer into in real time, and I could get the team to think about it. But I find it still siloed, I am afraid It is a fantastic effort to make the mental health team more accessible to us but sitting behind you know the faces that we see once a month is not enough capacity to be react when we want the system to react […]. I want a continuity there and I think patient needs a continuity.

General Practitioner (42)

d. Structure of Primary Care Networks (PCNs)

The structure of the Primary Care Networks (PCNs) was also identified as a barrier to building relationships and establishing knowledge exchange pathways between primary and mental healthcare. Interviewees explain that PCNs (each one comprised of a group of GP Practices) operate autonomously, making it challenging to address silos created between primary and mental healthcare. There is also variation described regarding the degree of engagement within PCNs, where some GPs can choose not to collaborate, for example, due to workload. Thus, the structure and internal diversity of PCNs may increase the difficulty of engaging with mental health professionals and liaison practitioners.

Ideally, I would like a lot more of my colleagues to participate. It’s something that I encourage them to do. The thing is, with staff limitations and time limitations, they can possibly only spare one colleague to take part in these discussions. Ideally, we’d like everyone to be involved but, sadly, there’s only, probably, provision for one person to be involved.

General Practitioner (41)

## 4 Discussion

Findings from this service evaluation suggest that the *set of boundary spanning processes and practitioner roles* implemented as part of the Peterborough Exemplar enables knowledge exchange and dissemination of expertise knowledge between primary and mental healthcare and, as a result, leads to optimisation of joint decisions about mental health treatment and care access. Nonetheless, partial engagement of GPs restrains full implementation; analysis identified reasons that potentially inhibit further engagement.

We found that boundary spanning inter‐organisational processes and professionals can improve the primary—secondary mental healthcare interface, by creating the necessary space for professionals from both parties to discuss patient cases and solutions for treatment. This is enabled by boundary spanning structured processes, that is, Virtual Clinics, which are available for GPs to access expertise of mental health professionals used to inform their decisions about patients' treatment. In this case, processes function as a facilitator for mental health professionals who act as boundary spanners. In a similar vein, boundary spanning processes also create opportunities for GPs with leadership roles to discuss operational barriers and service activity data with mental health professionals (i.e., in PCN‐PCMHS meetings), allowing for reviewing and jointly considering future service improvements. Here, both GPs and mental health professionals act as boundary spanners. As a result, boundary spanning processes create opportunities for GPs and mental health professionals to collaborate for improving care delivery and also facilitate the development of the system's learning capability by sharing lessons learnt from practice. This finding contributes to understanding the value boundary spanning structured processes add to the boundary spanning roles, as well as on enabling collaboration between physical and mental healthcare, by creating time and space for professionals to exchange knowledge and inform decisions.

Findings suggest that optimisation of decision making is facilitated by mental health professionals with boundary spanning responsibilities. Liaison practitioners who act as boundary spanners support the management of service users at a primary care level by offering mental health expertise to GPs to inform their decisions about service users' care pathways and medication. Boundary spanning roles in healthcare have been extensively studied [25], yet limited research has been conducted for the contribution of such roles to the interface between primary physical and secondary mental healthcare. Our findings propose that liaison practitioners with expertise on mental health conditions (e.g., personality disorders) can enable sharing of mental health knowledge and the identification of suitable treatment for mental health patients, including those with complex needs, within secondary specialist services as well as across the system.

Notably, we found that informal communication pathways were developed and used by GPs to reach out to practitioners with boundary spanning responsibilities and other mental health professionals, likely because of the positive impact of the aforementioned structured processes. This outcome was not identified as a target output of the intervention when designed, suggesting the organic growth of communication capabilities between primary and mental healthcare services, a key component to care integration [4]. Overall, our findings suggest that the set of boundary spanning roles and processes implemented increased clarity about care pathways and optimised service users' transition from primary to secondary mental healthcare.

Boundary spanning processes and roles did not address all inter‐organisational barriers. GPs' limited knowledge on available mental health services and accessibility processes indicates that the intervention did not reach the full cohort of local GPs, or perhaps more time is required for the intervention to fully penetrate in practice. Evaluation of complex interventions suggest that longer periods of implementation are required to assess their impact in care delivery [38]. Additional system factors may have impacted the implementation process of boundary spanning processes which we may not have captured.

GPs' professional views on the value and suitability of various mental health treatments and community support were also identified as a barrier that boundary spanners came across when collaborating. Indeed, literature suggests that barriers to inter‐organisational interface can be attributed to operational boundaries that may exist among organisations, as well as to diverse professional perspectives [38, 39]. Our findings align with studies on care integration proposing that addressing integration barriers should entail considering cultural differences, values and diverse professional approaches that pre‐exist within each organisation and lie beyond operational barriers [39]. Evaluation findings suggest that such soft barriers occurring in the primary healthcare—secondary mental healthcare interface remained an inhibiting factor in the collaboration between GPs and mental health professionals when seeking treatment solutions. Findings did not indicate whether difficulties with bridging divergent professional views could be attributed to limited boundary spanning skills, or whether more time was needed for boundary spanning to address professional culture barriers.

Lastly, GP participants argued that not all their needs were met regarding supported needed for mental health patients, while parts of the interventions were perceived as additional workload, proposing that those parameters were not fully considered by the Exemplar developers or maybe occurred following implementation. PCNs' structure as a barrier to implementation indicates that inter‐organisational boundaries may be caused by both primary and secondary care services pointing out that integration requires to jointly seek solutions that improve siloed working. This finding highlights the impact the wider inter‐organisational context can have on the implementation of boundary spanning interventions.

The main strength of this evaluation is that it adds valuable insight on the role of boundary spanners who are mental health specialists and on the positive impact the roles can have on the challenging interface between primary care and secondary mental healthcare. It also highlights wider system barriers than can restrain the contribution of boundary spanning roles. Findings also increase insight on the positive impact boundary spanning processes on facilitating the role of boundary spanners and enable knowledge exchange.

The evaluation presents certain limitations; Four GPs participated in qualitative interviews instead of eight initially planned, and one PCN Lead. Participating GPs interacted regularly with the PCMHS, allowing for in‐depth exploration of the interview questions. Also, services users were not interviewed, as the focus of the intervention was mainly on the addressing the barriers among professionals between primary and secondary care. Service users and their carers were involved in the development of the study design; regarding qualitative interviews, they participated in developing the interview guide.

## 5 Conclusion

The implemented set of boundary spanning roles and processes led to improved collaboration in parts of the community mental healthcare system where rigid organisational boundaries limit joined care, decision optimisation and development of sustainable relationships between the primary and mental healthcare. Findings from the service evaluation of the Peterborough Exemplar suggest that the implementation of boundary spanning practices enhance communication between primary and mental healthcare and, as a result, improve accessibility to services. Systematic and effective collaboration appears to also be dependent on the degree to which primary care professionals (i.e., GPs) are engaged, signalling the need for increasing system working in findings solutions for addressing care integration barriers. Findings from the service evaluation could be used to inform national policies on care integration and the development of local care integration pathways in community mental healthcare systems. The local organisational and inter‐organisational context should be considered and the impact it may have on the effectiveness and implementation of boundary spanning practices.

## Author Contributions

L.E. contributed to draughting the manuscript, data collection and analysis. J.M. contributed to the data collection process. A.P.W. and R.C. contributed to the study design and reporting of findings. J.J. and J.P. provided insight on the data analysis and reporting of findings. All authors contributed to draughting the manuscript and have approved the final manuscript.

## Ethics Statement

This evaluation was approved by the Quality Improvement Department of the Cambridgeshire and Peterborough NHS Foundation Trust (CPFT).

## Consent

Participants in qualitative interviews received a participant information sheet (PIS) and a consent form (CF) to participate in the study. Participants were informed that participation to interviews is anonymous, confidential, and that anonymised quotes will be included in published reports and academic papers.

## Conflicts of Interest

The authors declare no conflicts of interest.

## Data Availability

Qualitative data collected are not shared in public to mitigate risks of identification of participants. Anonymised quotes are included in the manuscript to support findings.

## References

[1] G. Thornicroft , T. Deb , and C. Henderson , “Community Mental Health Care Worldwide: Current Status and Further Developments,” World Psychiatry 15, no. 3 (2016): 276–286, 10.1002/wps.20349. 27717265 PMC5032514

[2] G. Thornicroft and M. Tansella , “The Balanced Care Model: The Case for Both Hospital‐ and Community‐Based Mental Healthcare,” British Journal of Psychiatry 202, no. 4 (2013): 246–248, 10.1192/bjp.bp.112.111377. 23549938

[3] C. Naylor , H. Taggart , and A. Charles , “New Models of Care in Mental Health NHS,”2017, https://www.kingsfund.org.uk/sites/files/kf/field/field_publication_file/MH_new_models_care_Kings_Fund_May_2017.pdf.

[4] C. Burke , J. Broughan , G. McCombe , R. Fawsitt , Á Carroll , and W. Cullen , “What Are the Priorities for the Future Development of Integrated Care? A Scoping Review,” Journal of Integrated Care 30, no. 5 (2021): 12–26, 10.1108/JICA-01-2021-0002.

[5] S. Baxter , M. Johnson , D. Chambers , A. Sutton , E. Goyder , and A. Booth , “The Effects of Integrated Care: A Systematic Review of UK and International Evidence,” BMC Health Services Research 18, no. 1 (2018): 350, 10.1186/s12913-018-3161-3. 29747651 PMC5946491

[6] C. Coughlan , N. Manek , Y. Razak , and R. E. Klaber , “How to Improve Care Across Boundaries,” BMJ 369 (2020): m1045, 10.1136/bmj.m1045. 32245848 PMC7190271

[7] P. Williams , “The Competent Boundary Spanner,” Public Administration 80, no. 1 (2002): 103–124, 10.1111/1467-9299.00296.

[8] NHS England, NHS Improvement, National Collaborating Central for Mental Health . The Community Mental Health Framework for Adults and Older Adults (2019).

[9] P. Das , C. Naylor , and A. Majeed , “Bringing Together Physical and Mental Health Within Primary Care: A New Frontier for Integrated Care,” Journal of the Royal Society of Medicine 109, no. 10 (2016): 364–366, 10.1177/0141076816665270. 27729592 PMC5066536

[10] B. Baird , C. Wickens , and S. Zearmal , Primary Care Networks (PCNs) Explained (King’s Fund, 2024), https://www.kingsfund.org.uk/insight‐and‐analysis/long‐reads/primary‐care‐networks‐explained.

[11] C. Naylor , A. Bell , B. Baird , A. Heller , and H. Gilburt , Mental Health and Primary Care Networks: Understanding the Opportunities, (2020), https://assets.kingsfund.org.uk/f/256914/x/a0ccc65526/mental_health_and_primary_care_network_2020.pdf.

[12] B. Baird , A. Charles , M. Honeyman , D. Maguire , and P. Das , Understanding Pressures in General Practice, (2016), https://www.kingsfund.org.uk/sites/default/files/field/field_publication_file/Understanding‐GP‐pressures‐Kings‐Fund‐May‐2016.pdf.

[13] C. McCartan , T. Adell , J. Cameron , et al., “A Scoping Review of International Policy Responses to Mental Health Recovery During the COVID‐19 Pandemic,” Health Research Policy and Systems 19, no. 1 (2021): 58, 10.1186/s12961-020-00652-3. 33823855 PMC8022299

[14] K. Garratt , Mental Health Policy and Services in England, (2024), https://commonslibrary.parliament.uk/research‐briefings/cbp‐7547/.

[15] H. Alderwick and J. Dixon , “The NHS Long Term Plan,” BMJ 364 (2019): l84, 10.1136/bmj.l84. 30617185 PMC6350418

[16] A. Dowrick , M. Kelly , and G. Feder , “Boundary Spanners: Negotiating Connections Across Primary Care and Domestic Violence and Abuse Services,” Social Science & Medicine 245 (2020): 112687, 10.1016/j.socscimed.2019.112687. 31759249

[17] C. Wallace , J. Farmer , and A. McCosker , “Boundary Spanning Practices of Community Connectors for Engaging ‘Hardly Reached’ People in Health Services,” Social Science & Medicine 232 (2019): 366–373, 10.1016/j.socscimed.2019.05.034. 31132544

[18] M. B. A. Hatch , J. K. Parrish , S. S. Heppell , et al., “Boundary Spanners: A Critical Role for Enduring Collaborations Between Indigenous Communities and Mainstream Scientists,” Ecology and Society 28, no. 1 (2023): art41, 10.5751/ES-13887-280141.

[19] J. W. Neal , Z. P. Neal , and B. Brutzman , “Defining Brokers, Intermediaries, and Boundary Spanners: A Systematic Review,” Evidence & Policy 18, no. 1 (2022): 7–24, 10.1332/174426420X16083745764324.

[20] A. T. Bednarek , C. Wyborn , C. Cvitanovic , et al., “Boundary Spanning at the Science–Policy Interface: The Practitioners’ Perspectives,” Sustainability Science 13, no. 4 (2018): 1175–1183, 10.1007/s11625-018-0550-9. 30147800 PMC6086300

[21] W. Stephens , R. van Steden , and L. Schoonmade , “Boundary Spanning in Local Governance: A Scoping Review,” Administration & Society 56, no. 2 (2024): 99–144, 10.1177/00953997231219262.

[22] P. Williams , “We Are All Boundary Spanners Now?,” International Journal of Public Sector Management 26, no. 1 (2013): 17–32, 10.1108/09513551311293417.

[23] Y. Liu and K. E. Meyer , “Boundary Spanners, HRM Practices, and Reverse Knowledge Transfer: The Case of Chinese Cross‐Border Acquisitions,” Journal of World Business 55, no. 2 (2020): 100958, 10.1016/j.jwb.2018.07.007.

[24] A. P. J. Schotter , R. Mudambi , Y. L. Doz , and A. Gaur , “Boundary Spanning in Global Organizations,” Journal of Management Studies 54, no. 4 (2017): 403–421, 10.1111/joms.12256.

[25] L. Nasir , G. Robert , M. Fischer , I. Norman , T. Murrells , and P. Schofield , “Facilitating Knowledge Exchange Between Health‐Care Sectors, Organisations and Professions: A Longitudinal Mixed‐Methods Study of Boundary‐Spanning Processes and Their Impact on Health‐Care Quality,” Health Services and Delivery Research 1, no. 7 (2013): 1–170, 10.3310/hsdr01070. 25642564

[26] J. C. Long , F. C. Cunningham , and J. Braithwaite , “Bridges, Brokers and Boundary Spanners in Collaborative Networks: A Systematic Review,” BMC Health Services Research 13, no. 1 (2013): 158, 10.1186/1472-6963-13-158. 23631517 PMC3648408

[27] Gilburt H. Supporting Integration Through New Roles and Working Across Boundaries. (2016).

[28] J. D. Alexander , “Public–Private Partnerships, Boundary Spanners and the Boundary Wall in the English National Health Service,” Journal of Health, Organisation and Management 38, no. 5 (2024): 662–681, 10.1108/JHOM-01-2023-0002. 39008089

[29] L. Efstathopoulou , G. Jagger , J. Mackenzie , et al., “The Peterborough Exemplar: A Protocol to Evaluate the Impact and Implementation of a New Patient‐Centred, System‐Wide Community Mental Healthcare Model in England,” Health Research Policy and Systems 20, no. 1 (2022): 16, 10.1186/s12961-022-00819-0. 35123500 PMC8817469

[30] P. Craig , P. Dieooe , S. Macintyre , S. Michie , I. Nazareth , and M. Petticrew , “Developing and Evaluating Complex Interventions: Folloving Considerable Development in the Field Since 2006, MRC and NIHR Have Jointly Commissionned an Update of This Guidance to Be Published in 2019,” Medical Research Council (2019): 1–39.

[31] A. Tong , P. Sainsbury , and J. Craig , “Consolidated Criteria for Reporting Qualitative Research (COREQ): A 32‐Item Checklist for Interviews and Focus Groups,” International Journal for Quality in Health Care 19, no. 6 (2007): 349–357, 10.1093/intqhc/mzm042. 17872937

[32] C. A. B. Warren , “Qualitative Interviewing,” in Handbook of Interview Research (SAGE Publications Inc., 2016), 83–102, 10.4135/9781412973588.n7.

[33] C. McGrath , P. J. Palmgren , and M. Liljedahl , “Twelve Tips for Conducting Qualitative Research Interviews,” Medical Teacher 41, no. 9 (2019): 1002–1006, 10.1080/0142159X.2018.1497149. 30261797

[34] L. A. Palinkas , S. M. Horwitz , C. A. Green , J. P. Wisdom , N. Duan , and K. Hoagwood , “Purposeful Sampling for Qualitative Data Collection and Analysis in Mixed Method Implementation Research,” Administration and Policy in Mental Health and Mental Health Services Research 42, no. 5 (2015): 533–544, 10.1007/s10488-013-0528-y. 24193818 PMC4012002

[35] V. Braun and V. Clarke , “Using Thematic Analysis in Psychology,” Qualitative Research in Psychology 3, no. 2 (2006): 77–101, 10.1191/1478088706qp063oa.

[36] QSR International . “NVivo Qualitative Data Analysis Software,”.

[37] NHS Health Research Authority . “NHS Health Research Authority: Accessing Study Support and Advice Services,”.

[38] N. Curry , M. Harris , L. H. Gunn , et al., “Integrated Care Pilot in North‐West London: A Mixed Methods Evaluation,” International Journal of Integrated Care 13, no. 3 (2013), 10.5334/ijic.1149. PMC3807631 24167455

[39] N. Goodwin , “Understanding and Evaluating the Implementation of Integrated Care: A ‘Three Pipe’ Problem,” International Journal of Integrated Care 16, no. 4 (2016), 10.5334/ijic.2609. PMC5354212 28316558

